# Low—Permittivity Copolymerized Polyimides with Fluorene Rigid Conjugated Structure

**DOI:** 10.3390/ma14216266

**Published:** 2021-10-21

**Authors:** Xiaodi Dong, Mingsheng Zheng, Baoquan Wan, Xuejie Liu, Haiping Xu, Junwei Zha

**Affiliations:** 1 School of Chemistry and Biological Engineering, University of Science & Technology Beijing, Beijing 100083, China; dongll1997@126.com (X.D.); baoquan_w@126.com (B.W.); b20190388@xs.ustb.edu.cn (X.L.); 2 Shanghai Engineering Research Center of Advanced Thermal Functional Materials, Shanghai Polytechnic University, Shanghai 201209, China; hpxu@sspu.edu.cn; 3 Beijing Advanced Innovation Center for Materials Genome Engineering, University of Science & Technology Beijing, Beijing 100083, China

**Keywords:** copolyimide, low permittivity, thermal stability, conjugated structure

## Abstract

As the miniaturization of electronic appliances and microprocessors progresses, low-permittivity interlayer materials are becoming increasingly important for their suppression of electronic crosstalk, signal propagation delay and loss, and so forth. Herein, a kind of copolyimide (CPI) film with a “fluorene” rigid conjugated structure was prepared successfully. By introducing 9,9-Bis(3-fluoro-4-aminophenyl) fluorene as the rigid conjugated structure monomer, a series of CPI films with different molecular weights were fabricated by in situ polymerization, which not only achieved the reduction of permittivity but also maintained excellent thermodynamic stability. Moreover, the hydrophobicity of the CPI film was also improved with the increasing conjugated structure fraction. The lowest permittivity reached 2.53 at 10^6^ Hz, while the thermal decomposition temperature (*T*_d5%_) was up to 530 °C, and the tensile strength was ≥ 96 MPa. Thus, the CPI films are potential dielectric materials for microelectronic and insulation applications.

## 1. Introduction

Polyimides (PIs) have been widely applied in packaging and insulation fields due to their excellent mechanical properties, ease of processing, temperature resistance, and so forth [1,2,3,4,5]. However, the high permittivity (*ε* = 3.0 ~ 3.4, *f* = 10^2^ Hz ~ 10^6^ Hz) of conventional polyimides (PIs) cannot meet the current demands in highly integrated and miniaturization electronic and electrical equipment [6], which severely restricts their further applications. Therefore, it is urgent to design and develop new types of PIs with low permittivity. In order to obtain the low-permittivity polymer materials, two effective strategies are generally considered— (1) reducing the molar polarization rate of the material; (2) introducing air into the polymer matrix (*ε*_air_ ≈ 1.0) [7,8,9,10,11,12]. For example, Zhang et al. reported that a kind of PI fabricated by fluorinated polybenzoxazole derived from the benzoxazine monomer possessed the combined excellent properties of facile synthesis, easy processability, and low permittivity [13]. Specifically, the permittivity of fluorinated polybenzoxazole had a range of 2.19 ~ 2.42 from 1 Hz to 10^6^ Hz. Chen et al. applied two diamine monomers containing triphenylmethane side groups with different substituents and prepared a series of FPIs from them with dianhydride 6FDA [14]. It was found that the permittivity of the FPI film was reduced to 2.25 (*f* = 10^6^ Hz). Compared to ordinary PI film, introducing a porous structure can significantly suppress the permittivity of polyimide film [15]. Kourakata et al. successfully prepared porous polyimide films with permittivity of 1.50 (*f* = 10^6^ Hz) using silica particles of different particle sizes as templates [16]. Yang et al. used the high-internal phase Pickering emulsification (HIPPE) process and high-temperature thermocompression to prepare a series of porous BN/PI composites [17]. The different content of BN directly affects the porous morphology of the polyimide composites. When the mass fraction of BN was 20 wt %, the permittivity of the porous PI reached 2.12, and the dielectric loss was close to zero (<0.002) at a high frequency. Considering the actual application environment, low-permittivity polyimides should also have excellent hydrophobicity. Due to its high permittivity (*ε* ≈ 80), water has a significant influence on the dielectric properties of polyimides [18]. Additionally, in the microelectronics industry, the absorption of water between the interlayer dielectrics is an important issue for microelectronic devices because the absorbed water will obviously enhance the conductivity of the interlayer dielectrics and damage the performance of microelectronic devices [2,11,19,20,21,22]. Dong et al. prepared porous polyimide films using 6FDA and ODA [23]. As a consequence of the internal microporous structure, its permittivity reached 1.91. When the relative humidity was 70%, the hydrophobic angle of the film was 161°, which had excellent hydrophobic properties. Although fluorinated or porous PIs could reach relatively low permittivity, undesirable weakened thermal and mechanical properties hinder their practical applications [1,24]. Moreover, the porousness usually enhances the water absorption of the polyimide films, which has a negative impact on subsequent processing procedures and industrial applications.

In this work, a rigid conjugated network according to the intrinsic molecular structure of polyimide was designed and fabricated to improve all of the dielectric, thermal, mechanical, and hydrophobic properties. A rigid conjugated structure could not only significantly restrain the intramolecular chain movement but also reduce the polarization, which will exhibit satisfactory dimensional stability, low permittivity, and excellent mechanical properties. In this study, the co-polyimides (CPIs) were prepared via in situ copolymerization using biphenyl-3,3′,4,4′-tetracarboxylic dianhydride (BPDA), 1,4-Bis(4-amino-2-trifluoromethylphenoxy) benzene (6FAPB), and 9,9-Bis(3-fluoro-4-aminophenyl) fluorene (FFDA) as comonomers. BPDA can impart many excellent properties, such as high-temperature stability, high glass-transition temperatures, and better processability to polyimides [25]. The fluorine atoms of 6FAPB are conducive to reducing the polarity of polyimide molecular chains, and FFDA contains both “fluorine” atoms and a rigid conjugated fluorene structure. The successful combination of rigid conjugated structures, flexible ether bonds, and fluorine atoms in the polyimide skeleton not only reduced the dielectric properties of the polyimide but also maintained excellent mechanical and thermal properties.

## 2. Materials and Methods

### 2.1. Materials

Biphenyl-3,3′,4,4′-tetracarboxylic dianhydride (BPDA) was purchased from Beijing Honghu United Chemical Products Co. Ltd., Beijing, China and purified via sublimation under reduced pressure for 24 h. 9,9-Bis(3-fluoro-4-aminophenyl) fluorene (FFDA) was purchased from Beijing Huawei Ruike Chemical Co. Ltd., Beijing, China, 1,4-Bis(4-amino-2-trifluoromethylphenoxy) benzene (6FAPB) was obtained from Beijing Yinuokai Technology Co., Ltd., Beijing, China, and N, N-dimethylacetamide (DMAC) was provided by Sino Pharm Group Chemical Reagent Co. Ltd., Shanghai, China.

### 2.2. Methods

**Figure 1:**
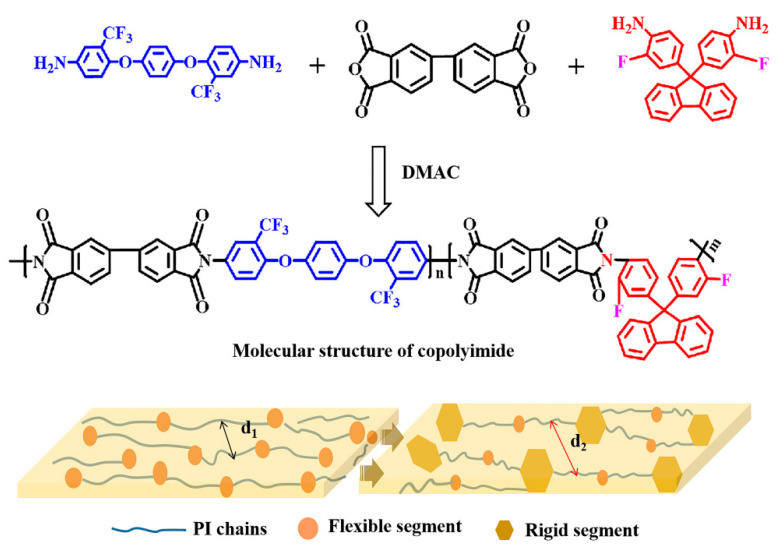
Molecular structure and schematic diagram of the microstructures of the CPI films.

A series of CPIs containing different molecular structures were synthesized, as shown in Figure 1. The molar ratios of 6FAPB and FFDA were 10:0, 8:2, 6:4, and 5:5, and the corresponding films were named PI-0, CPI-1, CPI-2, and CPI-3, respectively. Specifically, according to the molar ratios above, the 6FAPB, FFDA, and DMAC were added to a 50 mL three-necked flask with constant stirring to achieve the complete dissolution of the diamines in the nitrogen atmosphere. Then, a certain amount of BPDA was added, and the solution was stirred for 12 h at room temperature. After that, the polyamide acid (PAA) solution was poured onto a glass plate using a doctor blade to form copolymerized films, which were heated at 80 °C for 2 h and at 100 °C for 1 h in a vacuum oven. Finally, a three-stage gradient heating process was required to achieve the complete thermal imidization (150 °C for 1 h, 200 °C for 1 h, and 300 °C for 1 h, respectively) to obtain the CPI films, and the thicknesses of the CPI films were carefully controlled to about 12 ± 1 µm.

### 2.3. Characterization

Attenuated total reflectance Fourier-transform infrared spectroscopy (ATR-FTIR, BRUKER TENSOR 27 Spectrometer) was applied to detect the molecular structures of the CPI films. The morphology of the surfaces of the CPIs was studied using an atomic force microscopy (AFM) system (Dimension Icon, Bruker, Billerica, MA, USA). The X-ray diffraction (XRD) was measured on a Bruker D8 diffractometer. Differential scanning calorimetry (DSC) of ~5 mg samples was performed using a NETZSCH thermal analyzer (DSC 60) at a heating rate of 10 °C/min from 50 to 450 °C. A thermal gravimetric analyzer (TGA, SDT Q600) was applied to test the thermostability of the CPI films at a heating rate of 10 °C/min from 30 °C to 900 °C in the nitrogen atmosphere. For the measurement of mechanical properties, firstly, the CPI films were cut into splines with a length of 4 cm and a width of 1 cm, and a tensile testing machine (ESM303) was applied with a tensile speed of 13 mm/min at room temperature; each sample was tested at least five times. For the measurement of the dielectric properties, copper electrodes with diameters of 4 mm were firstly deposited on both sides using the high-vacuum resistance evaporation coating machine (Beijing Technol Science Co., Ltd., Beijing, China, ZHD-300). Then, the films coated with the copper electrodes were tested in a precision impedance analyzer (Agilent 4294A) in a range of 10^2^ Hz–10^7^ Hz at 500 mV and room temperature. Contact angle images were captured by an OCA20 device (Dataphysics, Filderstadt, Germany) with 5 μL water droplets (Milli-Q, 18.2 MΩ/cm) at ambient temperature.

## 3. Results and Discussion

### 3.1. Morphology of the CPI Films with Various Copolymerization Units

**Figure 2:**
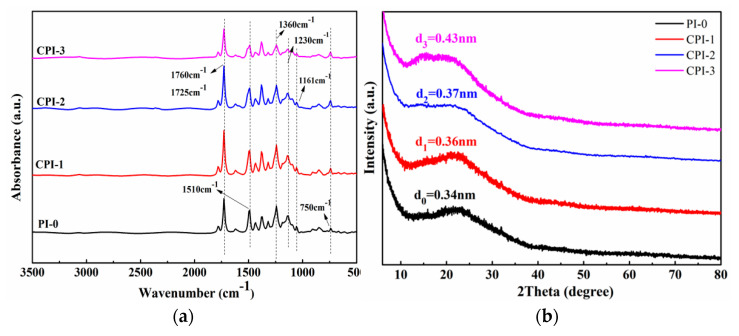
(**a**) ATR—FIR spectra and (**b**) XRD of the CPI films.

As shown in Figure 2a, all of the CPI films exhibited characteristic absorption bands of the imide ring near 1725 cm^−1^ and 1760 cm^−1^. The 1510 cm^−1^ was attributed to the stretching vibration of the benzene ring in the CPI molecule [1,26]. The C-N-C stretching vibration in the imide ring could be observed at about 1360 cm^−1^ and 750 cm^−1^, while the peaks near 1230 cm^−1^ and 1161 cm^−1^ were caused by the stretching vibration of the C-F [27]. The results indicate that the rigid fluorene structure was successfully introduced into the PI molecular chain. To explore the internal aggregation structure of the CPI molecule, the morphology of the CPI films was also analyzed by XRD. Specifically, we calculated the diffraction angle corresponding to each polyimide through XRD, and the relationship between the diffraction angle and *d*-spacing could be established through the Bragg equation [25], as follows:

2*d*sinθ = *n*λ, (1)

where *d* is the chain space between the molecules, and θ is the diffraction angle (λ = 0.15406 nm, *n* = 1). Equation (1) can be used to calculate the *d*-spacing of different PIs. Generally, the *d*-spacing reflects the average distance between segments of the polymer chain [28,29,30]. As shown in Figure 2b, a broad peak between 20° and 25° was observed in each polyimide, which indicated that the prepared PIs were amorphous. However, it is worth noting that the traditional PI had a higher diffraction peak at about 24°, while the diffraction peak shifted to a lower degree with the introduction of the copolymer unit, which indicates wider chain spacing with the increase of the “fluorene” structure content. The corresponding *d*-spacing is 0.34 nm for PI-0, 0.36 nm for CPI-1, 0.37 nm for CPI-2, and 0.43 nm for CPI-3, which indicates higher free volume in the CPI films [6].

### 3.2. Thermal and Mechanical Properties of the CPI Films

**Figure 3:**
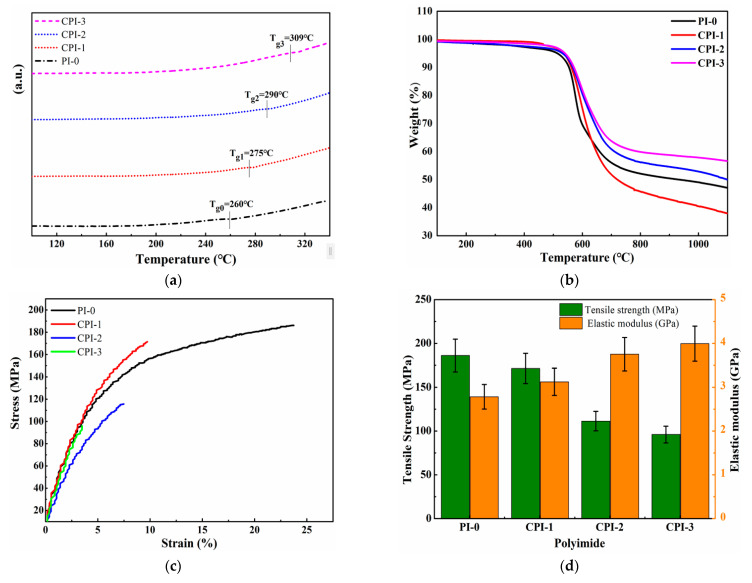
(**a**) DSC, (**b**) TGA, and **(c**) stress–strain curve of the CPI films. (**d**) Histogram of tensile strength and elastic modulus.

The thermostability of low-permittivity materials is considered one of the key parameters for high-density electronic and electrical equipment because the preparation of electronic components usually requires a high-temperature treatment [31,32]. The 5% weight loss (*T*_d5%_) and glass transition temperature (*T*_g_) are important factors for the evaluation of the thermostability of the materials. Here, a rigid conjugated structure was introduced into the molecular structure of the PI to improve the thermal stability. As shown in Figure 3a, the *T*_g_ values of the CPI films were approximately recorded in the range of 275 ~ 305 °C, which was higher than that of normal PI-0 film (260 °C). Meanwhile, the thermal decomposition rates of the CPI films were also improved. The *T*d_5%_ of the CPI films were up to 550 °C, as shown in Figure 3b. No decomposition was observed at 200–300 °C, which means that the PAA had been completely imidized and no amide moieties existed [23]. From the perspective of the molecular structure, due to the greater rigidity of the “fluorene ring” itself, the presence of the fluorene ring would reduce the thermal decomposition rates of the CPI films and increase the residual mass fraction. In addition, the conjugated structure between the fluorene ring, benzene ring, and the imide ring prevented the rotation and conformational changes of the molecular chain, which was also beneficial to improve the thermal stability of the CPI films.

To investigate the influence of FFDA on the mechanical properties of the CPI films, the tensile properties (tensile strength, elongation at break, and elastic modulus) of the CPI films were measured, as shown in Table 1. The establishment of the rigid conjugated structure slightly reduced the mechanical properties of the CPI films, but they still maintained high tensile strength and elastic modulus, which were 96 c 172 MPa and 3.13–4.00 GPa, respectively. As the rigidity of the polyimide molecular chain increased, the elongation at break of the CPI films decreased to 3.48% ~ 9.43%.

### 3.3. Dielectric Properties of the CPI Films

**Figure 4:**
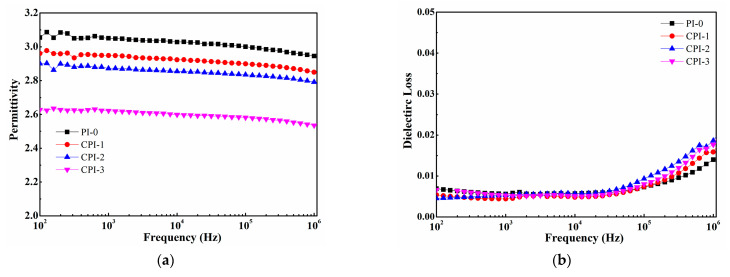
(**a**) Permittivity and (**b**) dielectric loss of the CPI films.

The dependence of the permittivity and loss of the CPI films on frequency is shown in Figure 4. It can be seen that the permittivities of the CPI films significantly decreased with the increasing FFDA contents. The permittivity of the CPI-3 film could be as low as 2.53 at 10^6^ Hz. The reason for the superior dielectric properties of the CPI films could be explained by Clausius–Mosotti equation, as follows:

(2) ε=1+2 (PmVm)1 - (PmVm)

where *ε*, *P*_m_, and *V*_m_ are the permittivity, molar polarizability, and volume of the atomic group, respectively. According to Equation (2), the increase in *V*m or the decrease in *P*m would cause the permittivity to decrease. Therefore, it is a guide to design and synthesize low-permittivity PIs with larger molar free volume or low molar polarizability. In this work, for the CPI film, on the one hand, the existence of the bulky fluorene structure brought about less efficient chain packing and more free volume, and the rigid conjugate structure limited the mobility of the dipole under an applied electric field. On the other hand, the strong electronegativity of fluorine also resulted in the C-F bonds with lower polarizability [2,33]. Therefore, the two reasons discussed above led to the decrease in the permittivity of the CPI films. Additionally, as shown in Figure 4b, the dielectric loss of the CPI films remained relatively low (~6 × 10^−3^) at a low frequency, while exhibiting an upward trend at a range of 10^5^ ~ 10^6^ Hz due to the polarization relaxation [34].

### 3.4. Hydrophobicity Properties of the CPI Films

**Figure 5:**
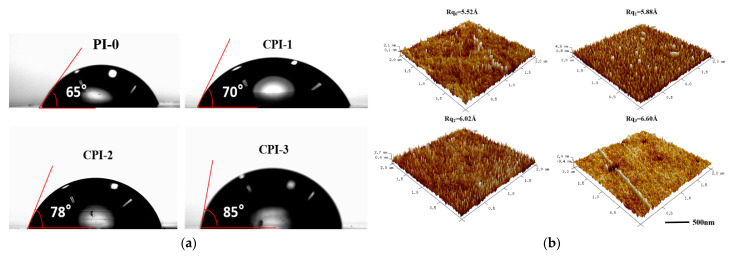
(**a**) Contact angles and (**b**) AFM images of the CPI films.

The surface hydrophobicity was characterized by the contact angle of a water droplet on the solid surface. Figure 5a shows the contact angle of the CPI films, which ranged from 70° to 85° ± 1.1°. It was indicated that the surfaces of the CPI films became more hydrophobic after the copolymerization. The decrease in water absorption might be ascribed to the increase in the proportion of FFDA, which increased the number of fluorine atoms in the polyimide backbone. Therefore, the hydrophobic properties of the CPI films inhibited the absorption of water. In addition, the surfaces of the CPI films are smooth and compact, and their surface roughness (Rq) changed slightly as the FFDA content increased, as shown in Figure 5b. This phenomenon revealed that the fact that the CPI films contained no nano-porous structure, and the realization of low dielectric properties was due to the large free volume in the amorphous region of the polyimide [6].

## 4. Conclusions

In summary, the as-fabricated CPI films were successfully prepared by BPDA, 6FAPB, and FFDA via a copolymerization reaction. Specifically, the CPI-3 film showed the lowest permittivity (the value of the permittivity was only 2.53 at 10^6^ Hz) and relatively low dielectric loss. Moreover, the CPI films showed a combination of outstanding comprehensive properties, such as enhanced mechanical properties (tensile strength of ≥ 96 MPa), outstanding dimensional stability, superior thermal stability (*T*_g_ ≥ 275 °C, *T*_d5%_ ≥ 550 °C), and excellent surface planarity. Thus, the desirable overall performances make this series of CPI films strong candidates for constructing the next generation of high-speed integrated circuits. The incorporation of cross-linkable side-groups will be an effective and convenient way to remarkably decrease the permittivity and improve the comprehensive properties of polyimides simultaneously, and such a modified strategy can also be extended to other polymer structures to acquire novel high-performance materials.

## Figures and Tables

**Table 1:** Thermal and mechanical properties of the CPI films.

Samples	*T*_d5%_ (°C)	*T*_d10%_ (°C)	Tensile Strength (MPa)	Percentage of Breaking Elongation (%)	Elastic Modulus (GPa)
PI-0	514	554	186.15	23.75	2.79
CPI-1	532	562	171.49	9.73	3.13
CPI-2	535	567	115.66	6.93	3.76
CPI-3	541	571	96.09	3.48	4.00

## Data Availability

The data presented in this study are available upon request from the corresponding author. The data are not publicly available due to the fact that they are also part of an ongoing study.

## References

[1] Bei R.X., Qian C., Zhang Y., Chi Z.G., Liu S.W., Chen X.D., Xu J.R., Aldred M.P. (2017). Intrinsic low dielectric constant polyimides: Relationship between molecular structure and dielectric properties. J. Mater. Chem. C.

[2] Zha J.W., Jia H.J., Wang H.Y., Dang Z.M. (2012). Tailored ultralow permittivity in high-performance fluorinated polyimide films by adjusting nanoporous characteristics. J. Phys. Chem. C.

[3] Meador M.A.B., Malow E.J., Silva R., Wright S., Quade D., Vivod S.L., Guo H.Q., Guo J., Cakmak M. (2012). Mechanically strong, flexible polyimide aerogels cross-linked with aromatic triamine. ACS Appl. Mater. Interfaces.

[4] Liu M.Y., Wang Y.X., Ji J.Q., Chang X.H., Xu Q., Liu X.Y., Qin J.Q. (2020). A facile method to fabricate the polyimide aerogels with controllable microstructure by freeze-drying. Mater. Lett..

[5] Zhu Z.X., Yao H.J., Wang F., Dong J.X., Wu K.D., Cao J.X., Long D.H. (2019). Fiber reinforced polyimide aerogel composites with high mechanical strength for high temperature insulation. Macromol. Mater. Eng..

[6] Liu Y.W., Qian C., Qu L.J., Wu Y.N., Zhang Y., Wu X.H., Bing Z., Chen W.X., Chen Z.Q., Chi Z.G. (2015). A bulk dielectric polymer film with intrinsic ultralow dielectric constant and outstanding comprehensive properties. Chem. Mater..

[7] Qiu G., Ma W., Wu L. (2020). Low dielectric constant polyimide mixtures fabricated by polyimide matrix and polyimide microsphere fillers. Polym. Int..

[8] Kurimoto M., Kato T., Yoshida T., Kato C., Suzuoki Y. (2019). Low permittivity epoxy nanoporous composites filled with hollow nanosilica. IET Nanodielectrics.

[9] Song N.N., Yao H.Y., Ma T.N., Wang T.J., Shi K.X., Tian Y., Zhang B., Zhu S.Y., Zhang Y.H., Guan S.W. (2019). Decreasing the dielectric constant and water uptake by introducing hydrophobic cross-linked networks into co-polyimide films. Appl. Surf. Sci..

[10] Gao F., Guo Y., Zhang K., Meng N., Szafran M. (2019). Microstructure and dielectric properties of sub-micron hollow sphere (Ba_0.6_Sr_0.4_) TiO_3_/PVDF composites. IET Nanodielectrics.

[11] Dong W., Guan Y., Shang D. (2016). Novel soluble polyimides containing pyridine and fluorinated units: Preparation, characterization, and optical and dielectric properties. RSC Adv..

[12] Cheng T.J., Lv G.P., Li Y.T., Yun H., Zhang L.F., Deng Y.M., Lin L.P., Luo X.J., Nan J.M. (2021). Low Dielectric Polyimide/Fluorinated Ethylene Propylene (PI/FEP) Nanocomposite Film for High-Frequency Flexible Circuit Board Application. Macromol. Mater. Eng..

[13] Zhang K., Han L., Froimowicz P., Ishida I. (2017). A smart latent catalyst containing o-trifluoroacetamide functional benzoxazine: Precursor for low temperature formation of very high Performance polybenzoxazole with low dielectric constant and high thermal stability. Macromolecules.

[14] Chen W.X., Zhou Z.X., Yang T.T., Bei R.X., Zhang Y., Liu S.W., Chi Z.G., Chen X.D., Xu J.R. (2016). Synthesis and properties of highly organosoluble and low dielectric constant polyimides containing non-polar bulky triphenyl methane moiety. React. Funct. Polym..

[15] Zhang Y., Yu L., Su Q., Zheng H., Huang H., Chan H.L.W. (2012). Fluorinated polyimide-silica films with low permittivity and low dielectric loss. J. Mater. Sci..

[16] Kourakataa Y., Onoderaa T., Kasaia H., Jinnaia H., Oikawaab H. (2021). Ultra-low dielectric properties of porous polyimide thin films fabricated by using the two kinds of templates with different particle sizes. Polymer.

[17] Yang K., Kang Y.Y., Ahn H.J., Kim D.G., Park N.K., Choi S.Q., Won J.C., Kim Y.H. (2020). Porous boron nitride/polyimide composite films with high thermal diffusivity and low dielectric properties via high internal phase Pickering emulsion method. J. Ind. Eng. Chem..

[18] Joseph A.M., Nagendra B., Surendran K.P., Gowd E.B. (2015). Syndiotactic polystyrene/hybrid silica spheres of POSS siloxane composites exhibiting ultralow dielectric constant. ACS Appl. Mater. Interfaces.

[19] Kim J., Kwon J., Kim M., Do J., Lee D., Han H. (2016). Low-dielectric-constant polyimide aerogel composite films with low water uptake. Polym. J..

[20] Yuan C., Wang J., Jin K., Diao S., Sun J., Tong J., Fang Q. (2014). Postpolymerization of functional organ siloxanes: An efficient strategy for preparation of low-k material with enhanced thermostability and mechanical properties. Macromolecules.

[21] Martin S.J., Godschalx J.P., Mills M.E., Shaffer II E.O. (2000). Development of a low-dielectric-constant polymer for the fabrication of integrated circuit interconnect. Adv. Mater..

[22] Lei X., Chen Y., Qiao M., Tian L., Zhang Q. (2016). Hyperbranched polysiloxane (HBPSi)-based polyimide films with ultralow dielectric permittivity, desirable mechanical and thermal properties. J. Mater. Chem. C.

[23] Dong F.P., Li H.W., Lu L.Y., Xiong Y.Z., Ha C.S. (2019). High-Tg porous polyimide films with low dielectric constant derived from spiro-(adamantane-2,9′(2′,7′-diamino)-fluorene). Appl. Polym..

[24] Meador M.A.B., Mcmillon E., Sandberg A. (2014). Dielectric and other properties of polyimide aerogels containing fluorinated blocks. ACS Appl. Mater. Interfaces.

[25] Ghosh A., Sen S.K., Banerjee S., Voit B. (2012). Solubility improvements in aromatic polyimides by macromolecular engineering. RSC Adv..

[26] Wu T.T., Dong J., Gan F., Fang Y.T., Zhao X., Zhang Q.H. (2018). Low dielectric constant and moisture-resistant polyimide aerogels containing trifluoromethyl pendent groups. Appl. Surf. Sci..

[27] Liu L.P., Lv F.Z., Li P.G., Ding L., Tong W.S., Chu P.K., Zhang Y.H. (2016). Preparation of ultra-low dielectric constant silica/polyimide nanofiber membranes by electrospinning. Compos. Part A Appl. Sci. Manuf..

[28] Gao H., Yorifujia D., Jiang Z.H., Ando S. (2014). Thermal and optical properties of hyperbranched fluorinated polyimide/mesoporous SiO_2_ nanocomposites exhibiting high transparency and reduced thermo-optical coefficients. Polymer.

[29] Zhuang Y.B., Seong J.G., Do Y.S., Jo H.J., Cui Z.L., Lee J., Lee Y.M., Guiver M.D. (2014). Intrinsically Microporous Soluble Polyimides Incorporating Tröger’s Base for Membrane Gas Separation. Macromolecules.

[30] Jiang Q.Y., Zhang W.H., Hao J.M., Wei Y.F., Mu J.X., Jiang Z.H. (2015). A unique “cage-cage” shaped hydrophobic fluoropolymer film derived from a novel double-decker structural POSS with a low dielectric constant. J. Mater. Chem. C.

[31] Volksen W., Miller R.D., Dubois G. (2010). Low dielectric constant materials. Chem. Rev..

[32] Huang W.P., Zeng F., Zhao J.Q., Zhu W.S., Hui L.S. (2008). Low dielectric constant polymer. Synth. Mater. Aging Appl..

[33] Park S.J., Cho K.S., Kim S.H. (2004). A study on dielectric characteristics of fluorinated polyimide thin film. J. Colloid Interface Sci..

[34] Sun M.Z. (2000). Fundamentals of Dielectric Physics.

